# Critical thinking in the age of generative AI: implications for health sciences education

**DOI:** 10.3389/frai.2025.1571527

**Published:** 2025-05-21

**Authors:** Waqar M. Naqvi, Rohini Ganjoo, Michael Rowe, Aishwarya A. Pashine, Gaurav V. Mishra

**Affiliations:** ^1^Department of Health Professions Education, Faculty of Interdisciplinary Sciences, Datta Meghe Institute of Higher Education and Research, Wardha, India; ^2^Department of Biomedical Laboratory Science, George Washington University, Washington, DC, United States; ^3^School of Health and Care Sciences, University of Lincoln, Lincoln, United Kingdom; ^4^Department of Cardiovascular and Pulmonary Physiotherapy, Career College, Bhopal, India; ^5^Department of Radiodiagnosis, Jawaharlal Nehru Medical College, Datta Meghe Institute of Higher Education and Research, Wardha, India

**Keywords:** generative artificial intelligence, cognitive capacity, augmented intelligence, healthcare sciences, large language models, critical thinking

## 1 Introduction

Generative artificial intelligence (genAI) systems are progressively transforming health science education and research by assisting clinicians in diagnosis and structuring specific intervention regimens (Mir et al., 2023). Moreover, these technologies serve educators in yielding simple concept-based educational modules tailored as per student's requirements and large language models (LLMs) and analogous models display the potential for automated streamlined literature reviews, prompt generation of interpretations and conclusions, and effortless drafting of manuscripts within seconds (Mir et al., 2023; Al Kuwaiti et al., 2023; Gupta et al., 2024) thereby, offering a potentially high level of convenience and efficiency. Furthermore, many researchers contend that genAI has the potential to completely automate research processes, including drafting proposals, analyzing data, and composing concluding reports (Almansour and Alfhaid, 2024; Preiksaitis and Rose, 2023). Hence, these merits indicate a prospective future for genAI, particularly in domains that demand processing of large scale data and iterative analyses.

However, despite these evident positive outcomes, concerns persist regarding the quality of AI-generated outputs, which may include inaccuracies in text reporting that contribute to misinformation, logical inconsistencies, outdated or unverified claims, and hallucinated references, all of which undermine the academic credibility of writing (Athaluri et al., 2023; Farrelly and Baker, 2023; Sittig and Singh, 2024). In addition, a lack of transparency in datasets exacerbates ethical challenges and biases (Norori et al., 2021). In an era characterized by the expedited advancement and refinement of genAI, it is necessary to critically evaluate whether these systems encourage critical thinking and human intelligence or subtly undermine them. This raises an important question: are we unintentionally relinquishing the cognitive abilities that have propelled scientific and clinical advancements, as healthcare professionals progressively integrate genAI into clinical and academic domains? The dilemma lies in balancing the efficiency of genAI with the preservation of essential human cognitive skills, such as critical thinking, ethical reasoning, and independent problem-solving. At its core, this dilemma focuses on how health professionals, medical trainees, and early career researchers apply outcomes produced by genAI as overreliance risk supporting passive dependence on algorithm produced outcomes in a context where time and cognitive capacity are consistently constrained. The expertise involving scientific accuracy, ethical judgment, and diagnostic reasoning are cultivated through proactive contribution by integrating knowledge in innovative and contextually relevant approaches, critically evaluating evidence, and grappling with uncertainty that genAI cannot substitute (Passerini et al., 2025; Shoja et al., 2023).

## 2 GenAI and the risk of cognitive complacency

The significance of the risk of cognitive complacency is emphasized globally, as industry and academia compete to implement genAI tools, and have rapidly accelerated publications related to AI, reflecting both enthusiasm and apprehension. However, medical professionals and researchers may demonstrate overdependence on genAI tools due to faster processing, thus, reducing opportunities for independent problem-solving and critical thinking (Shoja et al., 2023; Zhai et al., 2024). Additionally, in medical research where precision plays an important factor, underlying biases in data training of genAI can propagate false information (Norori et al., 2021). The inefficacy of plagiarism detection software to recognize text generated by genAI, undermines conventional ethical integrity measures as the output may be erroneously identified as genuine scholarly writing (Farrelly and Baker, 2023), though some exceptions exist (Elkhatat et al., 2023; Weber-Wulff et al., 2023). This attitude leads to a workforce adept at utilizing genAI but deficient in cognitive analytical competencies. Moreover, an evolving repository of research studies warns against excessive reliance on genAI for academic work by emphasizing that while genAI can generate complex answers to assignments, it may encourage students to trade critical thinking for speed, eroding the meta-cognitive skills essential to patient care and clinical reasoning (Fan et al., 2024; Eachempati et al., 2024).

However, the ecosystem of scholarly communication stands at a crossroads: will the next generation of scientists and clinicians accept findings derived from genAI uncritically, prioritizing output over insight, or will they develop the intellectual resilience necessary to critically evaluate the outcomes? In the healthcare industry, the capability to incorporate evolving evidence into public health policy, interpret subtle patient cues, and navigate cultural sensitivities is not exclusively governed by algorithms or encoded by pre-trained genAI models. This concern is particularly relevant because these higher-order cognitive functions necessitate emotional intelligence, nuances, and analytical judgment which are fundamental for fostering innovative and ethical healthcare in the future. The credibility of scientific research is defined by ethical considerations, peer evaluation, and reproducibility (Prager et al., 2019). However, negligence in critically evaluating genAI produced research risks eroding these fundamental principles. Therefore, to ensure research integrity, transparency, and mitigate overreliance on automated results, it is essential to establish and integrate genAI usage policies in academia (Athaluri et al., 2023).

## 3 GenAI as a tool for augmenting human intelligence

Prominent journals warns of a global “feedback loop” that risks reinforcing pre-existing knowledge patterns at the expense of transformative inquiry if genAI tools remain unmonitored (Kwong et al., 2024). Furthermore, a recent analysis elucidates that true intelligence is characterized by the ability to solve novel, previously un-encountered problems, rather than relying on recycling pre-existing solutions (Gignac and Szodorai, 2024). The discussion highlights conceptual clarity on the distinction between “intelligence” and “achievement” within both human and artificial domains (Gignac and Szodorai, 2024). Contemporary generative AI systems, extensively trained on existing datasets, may mimic expertise; however, they frequently lack demonstration of true intelligence, as their outputs are predominantly dependent on prior data exposure (Gignac and Szodorai, 2024). By consistently relying on familiar patterns, there is a risk of fostering intellectual complacency rather than encouraging original thoughts. This distinction is particularly significant in the health sciences, where true innovation emerges from confronting novel challenges, rather than revisiting established solutions.

GenAI should function as an assistive tool that enhances cognitive capabilities, embodying the concept of “augmented intelligence,” which emphasizes its role in complementing, rather than replacing human intellect and expertise (Monteith et al., 2024). It is crucial to strike a balance between human supervision and the efficiencies of genAI, cautioning that while genAI has the potential to optimize specific tasks, genuine depth, and innovation arise from the uniquely human cognitive abilities for critical reflection and rigorous interrogation (Sittig and Singh, 2024). Human centered AI operates as a two-dimensional framework that strategically balances automation with human control, aiming to enhance both technological reliability and human agency. This approach fosters the development of trustworthy, reliable, and safe AI-assisted computer programs while enhancing human self-efficacy, performance, creativity, and responsibility (Shneiderman, 2020). Promoting the ethical use of genAI includes key principles such as fairness, unbiased decision making, transparency in datasets, accountability, autonomy, and data privacy (Jobin et al., 2019; Habli et al., 2020).

Both academia and industry should be urged to collaboratively oversee the development of more advanced genAI, emphasizing that scientific rigor (transparency, and reproducibility), and critical peer review must govern the evolution of these technologies. Without such oversight, claims associated with AI's imminent artificial general intelligence risk go unchallenged, promoting a narrative of effortless problem-solving that sidesteps human contribution (Sittig and Singh, 2024). In contrast, maintaining rigorous standards and embedding critical thinking in genAI integration ensures that researchers and clinicians remain architects of their intellectual landscapes. If properly contextualized, genAI may become a powerful collaborator, rather than a technological crutch.

## 4 Institutional responsibility in AI adoption

**Figure 1:**
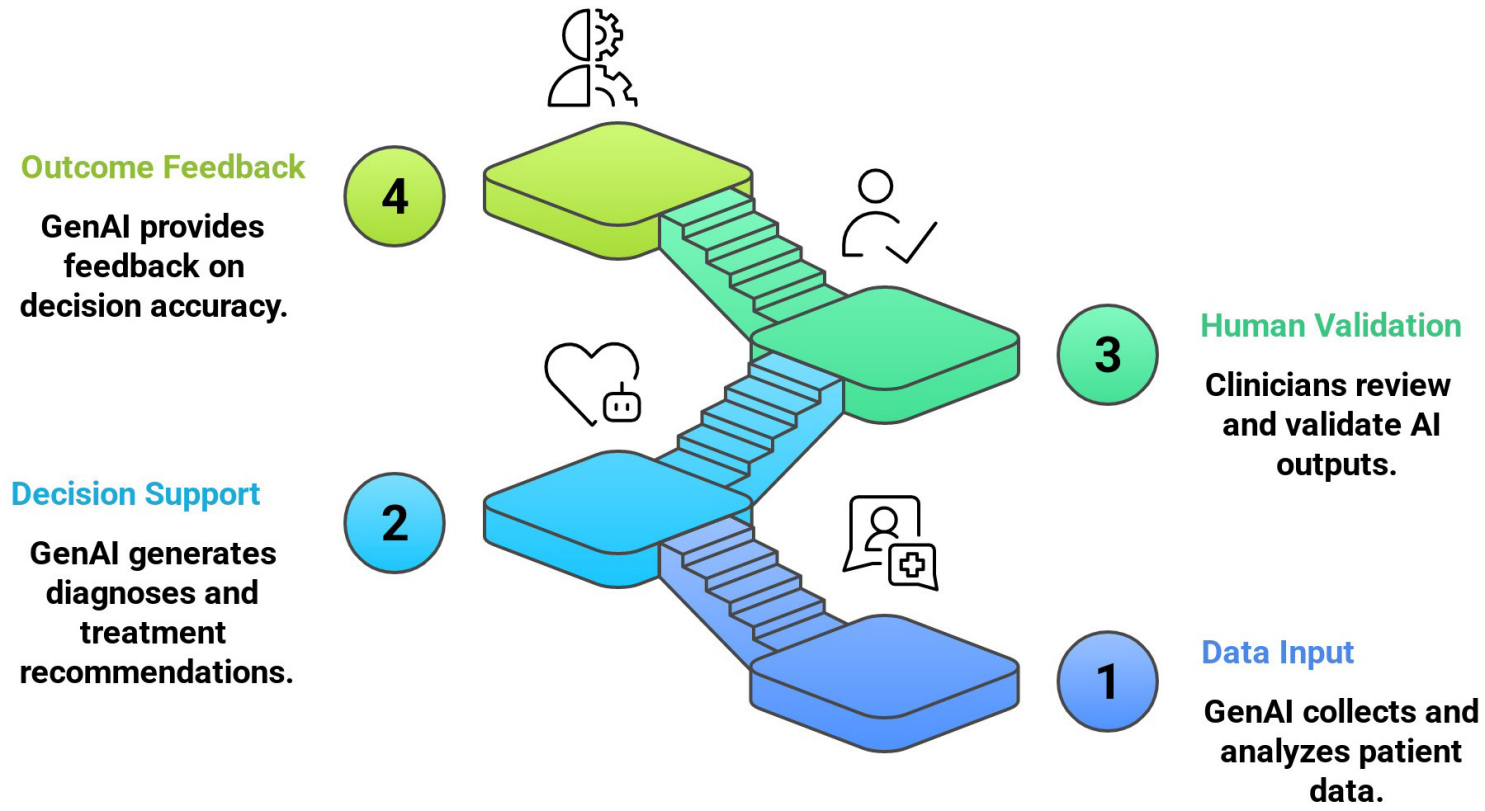
GenAI augmented clinical decision-making.

Graduate programs play a pivotal role in steering the ethical incorporation of genAI into health sciences. Medical education assisted by genAI mainly includes cloud computing, wearable devices, 5G, big data analysis, virtual reality, and the Internet of Things (Sun et al., 2023). Medical institutions must prioritize human supervision in research projects and clinical applications involving genAI (Janumpally et al., 2025) to ensure patient safety and ethical integrity (Figure 1). For instance, genAI is increasingly used for surgical training without direct patient involvement (Bhuyan et al., 2025). Western Michigan University exemplifies this by integrating over 100+ h of AI-simulation training in its medical curriculum, allowing students to engage in realistic patient scenarios under professor supervision (Bhuyan et al., 2025). Similarly, the University of Illinois College of Medicine successfully employs its AI in medicine (AI-Med) program, which cultivates critical appraisal skills required for assessing and implementing genAI in medical research. This initiative not only enhances proficiency in scientific writing and communication but also ensures the responsible dissemination of findings within the medical community (AI-MED, 2025). Congruently, the National University of Singapore mandatorily leverages undergraduate programs in bioinformatics and AI in medicine (Feigerlova et al., 2025). However, the overreliance on genAI poses significant risks. Inappropriate drug recommendations and misdiagnoses such as the inability to detect lesions or tumors, can be executed by genAI thus, endangering patient's lives (Saadat et al., 2024). For instance, despite insufficient accuracy, the widespread adoption of an externally validated AI sepsis prediction model flagged concerns leading to postponed intervention (Wong et al., 2021). These failures illustrate the critical need for human oversight and highlight the consequences of over-dependence on genAI. The key challenge encountered in genAI education involves curriculum standardization, due to resistance from some researchers and educators who lack the skills to detect the content produced by genAI or who rely on unreliable detection tools. This originates due to the inability of plagiarism detection software to differentiate between genAI produced content and human written text and due to a lack of knowledge and competency among health professionals regarding genAI technologies. For instance, tools such as PlagiarismCheck, CrossPlag, and Zero GPT often fail to detect genAI content due to the rapid evolution of these models, which progressively enhance their abilities to mimic human written text, making identification more challenging (Weber-Wulff et al., 2023; Elkhatat et al., 2023).

The curricula should therefore conceptualize genAI not as a shortcut but as a tool whose outputs demand scrutiny. Academic institutions should prioritize AI ethics in curriculum development, equipping future researchers and clinicians with the skills needed to critically engage with the content generated by genAI (Katznelson and Gerke, 2021; Bahroun et al., 2023). In fact, a study found that dental university instructors often mistook AI-generated reflections for student work, raising concerns about academic integrity and authorship (Brondani et al., 2024). Academic projects encouraging systematic synthesis of literature or research design through human cognitive abilities can allow students and trainees to identify biases, challenge assumptions, and detect conceptual gaps in conclusions derived from generative AI (Ganjoo et al., 2024). Moreover, healthcare professionals and students must be trained in AI literacy focusing on technical foundation, applications in real-world scenarios, the interaction between humans and genAI, critical thinking skills to differentiate genAI false claims and reality, strengthening of prompt engineering skills, and provide hands-on experience through case studies, and practical exercises, to apply genAI tools in simulated and real-life and to navigate its applications effectively (Charow et al., 2021; Walter, 2024; Sridharan and Sequeira, 2024). Incorporating assignments that require students to cross-check AI-generated information with reputable sources can foster critical thinking and responsible use of technology. This approach not only enhances students' research skills but also prepares them to navigate the ethical challenges associated with emerging AI technologies in their professional careers (Ganjoo et al., 2024; Masters et al., 2025). Integrating critical thinking into AI literacy courses ensures students grasp genAI's capabilities, limitations, ethical implications, and societal impact. By fostering analytical skills, educators help students become both technically proficient and ethically responsible. Furthermore, health professional educators must ensure the validation of genAI tools for accuracy and precision, personal data privacy, instructional strategies, assessment of teaching efficiency, learning outcomes, and feedback mechanisms for effective implementation of genAI-based educational programs (Feigerlova et al., 2025; Reddy, 2024; Shokrollahi et al., 2023).

GenAI should be framed as a tool for enhancing learning, not replacing traditional educational methodologies. Journals and academic bodies should enforce transparency in research submissions assisted by genAI. Specifically disclosing AI usage in manuscripts, ensuring human verification of AI-generated data, and maintaining ethical standards in publication practices are essential measures that preserve the credibility of scholarly communication in an AI-augmented academic environment (Koul, 2023; Yang et al., 2024). Encouraging transparency in the application of generative AI for both the researcher and publisher reinforces an environment where original reasoning is not overshadowed by the ease of prompt engineering.

## 5 Conclusion

By embracing the above-mentioned principles, we steer generative AI to augment human cognitive efforts, rather than replacing them. Instead of viewing efficiency and authenticity as competing values, we can align them using AI's processing power to handle repetitive tasks, while retaining human judgment for higher-level functions involving ethical reflection, contextual interpretation, and hypothesis generation. In doing so, we encourage a future in which technology catalyzes genuine innovation, inspiring thinkers to use genAI judiciously rather than dependently. It is essential to resist the allure of seamless outputs provided by generative AI that lack the precision and depth of the human intellect. The challenge is not to reject AI (quite the contrary) but to integrate it in ways that uphold the ethical, critical, and creative dimensions of health sciences. By doing so, we preserve the human spark that transforms data into discovery, complexities into clarity, and knowledge into wisdom.
